# Stimulus Novelty Energizes Actions in the Absence of Explicit Reward

**DOI:** 10.1371/journal.pone.0159120

**Published:** 2016-07-14

**Authors:** Raphael Koster, Tricia X. Seow, Raymond J. Dolan, Emrah Düzel

**Affiliations:** 1 Institute of Cognitive Neuroscience, University College London, London, WC1N 3AR, United Kingdom; 2 Wellcome Trust Centre for Neuroimaging, Institute of Neurology, University College London, London, WC1N 3BG, United Kingdom; 3 Otto von Guericke University Magdeburg, Institute of Cognitive Neurology and Dementia Research, D-39120, Magdeburg, Germany; 4 German Center for Neurodegenerative Diseases, D-39120, Magdeburg, Germany; ghent university, BELGIUM

## Abstract

Novelty seeking has been tied to impulsive choice and biased value based choice. It has been postulated that novel stimuli should trigger more vigorous approach and exploration. However, it is unclear whether stimulus novelty can enhance simple motor actions in the absence of explicit reward, a necessary condition for energizing approach and exploration in an entirely unfamiliar situation. In this study human subjects were cued to omit or perform actions in form of button presses by novel or familiar images. We found that subjects’ motor actions were faster when cued by a novel compared to a familiar image. This facilitation by novelty was strongest when the delay between cue and action was short, consistent with a link between novelty and impulsive choices. The facilitation of reaction times by novelty was correlated across subjects with trait novelty seeking as measured in the Tridimensional Personality Questionnaire. However, this li between high novelty-seeking and action facilitation was driven by trials with a long delay between cue and action. This prolonged time window of energization following novelty could hint at a mechanistic underpinning of enhanced vigour for approach and exploration frequently postulated for novelty seeking humans. In conclusion, we show that stimulus novelty enhances the speed of a cued motor action. We suggest this is likely to reflect an adaptation to changing environments but may also provide a source of maladaptive choice and impulsive behaviour.

## Introduction

Exploring novel options is an essential part of adaptive decision making behaviour [1,2], but is also linked to increased risk of addictive behaviour [3,4] and dopaminergic function [5,6]. The integration of novel stimuli in the choice process is thought to rely on the detection of previously unknown stimuli [7–9] and a bias on choice towards approaching and exploring the new stimuli [6].

fMRI studies in humans show that novelty signals are not only associated with activation in neocortical and limbic brain structures including the hippocampus [10–13], but also that stimulus novelty activates the substantia nigra/ventral tegmental area (SN/VTA) [6,14]. This is consistent with a functional anatomical model of a Hippocampus-SN/VTA loop in which hippocampal responses to novel stimuli activate the SN/VTA via an indirect pathway, through the ventral striatum and ventral pallidum [7,8,15]. More recently, an additional hippocampal-SN/VTA pathway has been detected originating in the CA3 subfield and relayed in the lateral septum [16]. Use of dopamine agonists in humans has also provided evidence consistent with novelty detection modulation by dopaminergic circuitry [17,18].

The dopaminergic SN/VTA’s central role in representing reward [19,20] and novelty detection has led to the hypothesis that novelty affects value guided decision making [21]. Previous results have shown that instrumental model-free learning is biased by stimulus novelty [6], an exploration bonus mediated by SN/VTA activation. A similar task in monkeys has shown that dopamine transporter blockade, resulting in enhanced dopaminergic activity, promotes novelty seeking when choosing whether to explore new options or exploit familiar ones [22].

Human approach behaviour is difficult to implement under experimental laboratory conditions. However, recent research indicates that button presses can be used as a model for approach [23–26] and contrasted with the omission of a button press as model for avoidance. Approach is linked to widespread activation of the basal ganglia, including the SN/VTA [23,24,27,28]. In this way dopamine release in response to novelty could benefit execution of motor actions [29] such as approach. The SN/VTA’s role in approach behaviour [23,24] led us to the question whether approach responses can be triggered by stimulus novelty in the absence of choice between rewarding options or even the absence of any reward at all. Such a finding would establish an effect of novelty on pure approach as a building block of impulsive behaviour, vigorous exploration, intrinsic motivation and potentially maladaptive choices.

To investigate whether novelty modulates approach tendencies, we adapted an experimental design in which Go or No Go responses are instructed by cue images [23,24,27]. In the experiment, the semantic category of novel and familiar stimuli determined whether or not an action was required. This allowed us to reveal congruency effects of novelty with action tendencies. If novelty affects motor tendencies, novelty related approach tendencies would lead to faster reaction times after seeing novel stimuli. In contrast, in the absence of such an invigorating effect of novelty, performance costs associated with the semantic categorization of the novel images should lead to longer reaction times. We also explored the possibility that the reaction time facilitation by novelty may depend on the temporal delay between a novel image and the required action. Greater reaction time facilitation after short delays would indicate a mechanism akin to impulsivity, whereas a delay independent facilitation would indicate sustained maintenance of motivation [30] akin to vigour and exploratory drive. We also hypothesized that novelty effects on reaction time would be modulated by individual differences in trait novelty seeking as measured by the Tridimensional Personality Questionnaire [31] and possibly vary with the length of the delay [6,32,33].

## Methods

### Participants

A group of 60 subjects participated in the study (Group 1: 23 males; age 18–20, mean: 22.6). Participants were recruited through a University College London participant pool, were self-reported right-handers, had normal or corrected to normal vision and were paid for their participation. The study was approved by the UCL local research ethics committee (PWB/ED/11-10-12b) and subjects gave informed written consent.

### Materials and Methods

**Fig 1:**
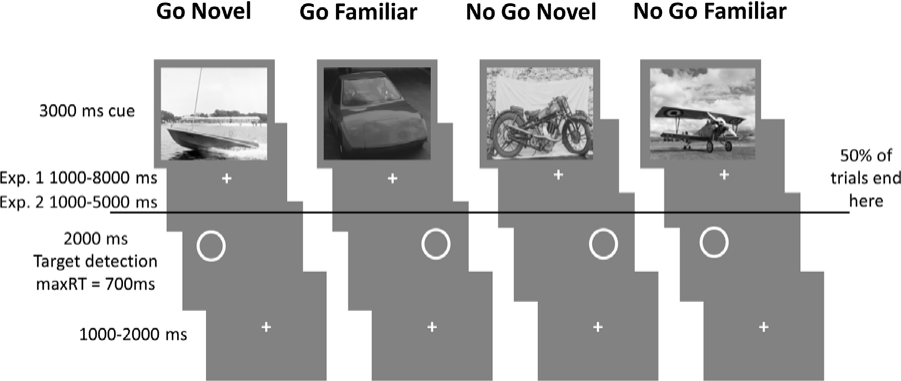
Experimental paradigm. Subjects were presented with 80 trials in each of four consecutive blocks. In each trial, an image from one of four categories (randomized across subjects) informed subjects about whether to press when a circle was displayed or to not respond (Go/No Go). In two conditions images were trial unique (Novel) and in two conditions four images within the category were repeated (Familiar). Subjects were pre-exposed to Familiar images during training. The cue was followed by a fixation period of varying length (1000–8000 ms in Experiment 1 and 1000–5000 ms in Experiment 2). Then, in 50% of the trials a circle was presented either on the left or the right instructing subjects to respond by pressing the left or right arrow key. This was followed by another fixation of variable length (1000–2000 ms).

The experiment (Fig 1) was adapted from Koster et al. 2015 [28] and consisted of four experimental runs, each run consisting of 80 trials. Each trial consisted of the two following events: presentation of a black and white square photograph (of one of four image categories: cars, boats, motorbikes, airplanes) for 3000 ms and the display of a circle for 2000 ms on either the left or right side of the screen. The cue and the response were separated by a fixation cross with a variable interval. The interval lasted between 1000–8000 ms in the long delay condition and 1000–5000 ms in the short delay condition. The response was then followed by a fixation cross for a variable interval of 1000-2000ms.

The image category instructed the participant whether on this particular trial it would be required to indicate the position of the circle with a keypress (Go) or to omit the response (No Go). Subjects were instructed that the Go response had to be entered in under 700 ms in order to be registered. Two of the image categories showed trial unique images (Novel), while the other two showed a repeating subset of four images. Additional to the repeats within the experiment itself, subjects were familiarized with the familiar images by exposure between training sessions and runs and the familiar images were used during the training session. The mapping of the image categories to experimental conditions was counterbalanced across participants. Each of the four experimental runs contained 20 trials per condition (Go Novel, Go Familiar, No Go Novel, No Go Familiar), half of which were aborted after the display of the image and did not require the Go/No Go response. Similarly, to not requiring action on half of the trials (No Go), aborting half the trials contributed to keeping subjects attentive and avoiding a habitual mode of constant responding. This makes the Go responses that are performed selective and appropriate. Aborting trials in the Go and No Go condition also makes the two conditions more comparable in terms of uncertainty and required attention due to unpredictable trial lengths, as well as making the paradigm more compatible with similar designs in the literature [23,24,28].

Subjects were told their performance would affect their payment but did not receive feedback after trials. To ensure that subjects learned the meaning of the image categories, subjects completed three runs of training. The first run consisted of 5 trials in which subjects were asked to indicate the position of the circle with a button press. The second run was one block in which no trial was aborted after image display, and subjects received feedback whether the response was correct. To ensure subjects were familiar with the manipulation by which half of the trials were aborted, another run of training consisted of a shortened version of an actual experimental run (approx. 10 minutes of total training time). During the training the images for the Novel condition were not trial unique but a repeating subset, that was not used in the actual experimental runs.

### Analysis

Analysis was conducted using Matlab 2010b, SPSS 19 and R. To analyse whether a novel cue image has a positive effect on RT overall, we entered RT of the two Go conditions in a 2 within (Novelty: Novel/Familiar) by 2 between (Delay length: short/long) ANOVA. The between group factor controls for the fact that two conditions contained a different range of delays between cue and action requirement. This analysis was repeated after nine subjects were removed for having a performance worse than 95% correct Go/No Go responses. Whether the Go/No Go responses were performed correctly was assessed with a 2x2 within (Novelty: Novel/Familiar and Action: Go/No Go) by 2 between (Delay length: short/long) ANOVA.

To test whether the delay between cue and action modulated the facilitation effect in both delay groups, the RT difference between novel and familiar images was binned (3 or 7 bins spanning over 1 second, respectively for the short and long delay group) and analysed in two one-way ANOVAs. To allow a more powerful analysis across both groups the bins were collapsed to include trials with a short (1000–2000 ms) vs longer (2000 and above) delay in a 2 within (short delay bin/long delay bin) by 2 between (Delay length: short/long) ANOVA. To investigate the effects further, one-sample t-tests were conducted in each bin (Bonferroni corrected for 7 comparisons).

The overall RT benefit of novelty was correlated with TPQ Novelty seeking scores. Correlation analyses with other TPQ scales have been conducted as exploratory analysis. To test the specificity of the correction with Novelty seeking the subscales Harm avoidance and Reward dependence were partialed out. The overall Novelty Seeking scale and its subscales were also correlated with the RT benefit for short and long delays. The strengths of the correlations were tested against each other with the psych library in R. Note that none of the exploratory and post-hoc correlations were corrected for multiple comparisons.

## Results

**Fig 2:**
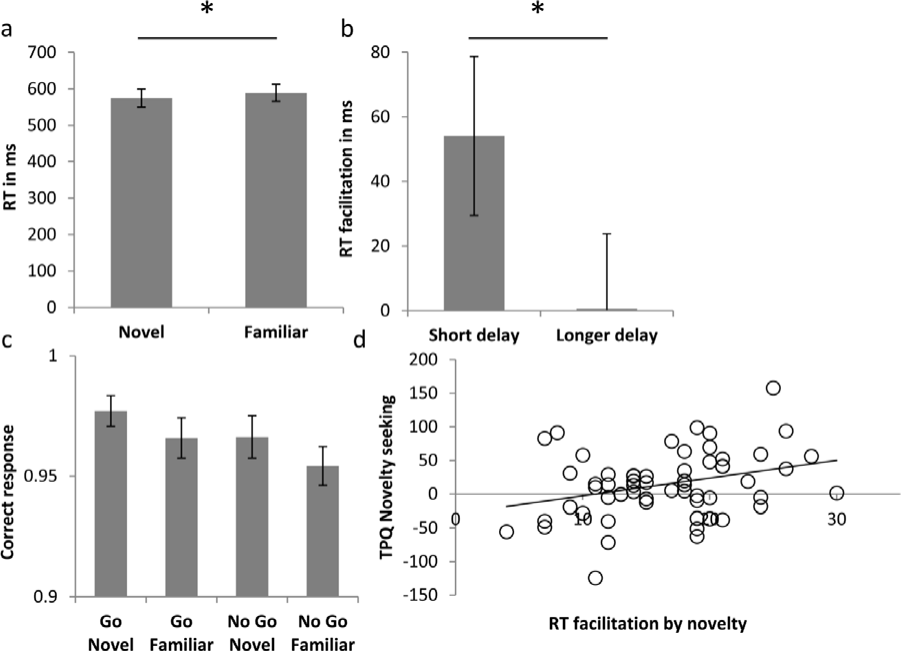
a. Reaction time in the Go conditions in both experimental samples. Key presses indicating the position of the circle (Fig 1) were significantly faster after subjects saw a novel image. b. This positive effect of novelty on the reaction time is significantly larger in trials in which the delay between the cue image and action is short (1000–2000 ms) compared to longer delays. c. Proportion of correct responses (Go or No Go) for each experimental condition. Novel images lead to more accurate responses. d. The overall benefit of novelty on reaction time is positively correlated across subjects with the TPQ Novelty Seeking scale. Subjects with a high Novelty Seeking score react faster after seeing novel images.

As displayed in Fig 2A, RTs for Go responses were facilitated following presentation of a novel cue image (mean+-standard deviation: 574.6+-194 vs 588.9+-183.1 ms; F(1,58) = 5.22,p = .024, partial η^2^ = .083) while there was no difference between delay conditions (F(1,58) = 1.45,p = .23, partial η^2^ = .024). Note that this result is a conservative estimate of the effect, as removing subjects with poor overall performance (lower than an arbitrary threshold of 95% correct responses (9 subjects), indicating possibly poor compliance with the instructions) resulted in a more robust facilitation by novelty (mean+-standard deviation: 534.6+-129.6 vs 553.9+-129.4 ms; F(1,49) = 9.441,p = .002, partial η^2^ = .162). Subjects were 96.6% correct in their responses on average and performed significantly more accurate after seeing a novel cue (F(1,58) = 9.3, p = .003, partial η^2^ = .14), independent of action or experimental group (all effects p>.1).

The RT facilitation by novelty revealed a statistical trend towards time bins differing from one another in the long delay group (F(6,174) = 2.273, p = .074) and a significant effect in the short delay group (F(3,87) = 3.19,p = .028, partial η^2^ = .099). To estimate the difference between the shortest and longer delays across both experimental groups, we analysed the delay in two bins (delays of 1000-2000ms vs. longer delays), revealing a significant difference (Fig 2B, F(1,58) = 6.12,p = .016, partial η^2^ = .095, no interaction with group: F(1,58) = .49,p = .83, partial η^2^ = .001, note that as this effect is calculated on the differences in RT between novel and familiar stimuli, it is equivalent to the interaction term between the factors of novelty and delay time). Analysing the RT facilitation in each individual time bin revealed a significant effect in the 1000-2000ms bin (t(59) = 3.26, p = .014, Bonferroni corrected for 7 comparisons; note that the time bin 4000-5000ms is significant on an uncorrected threshold t(59) = 2.38, p = .024).

The overall RT facilitation by novelty significantly correlated with the Novelty Seeking scale of the TPQ (Fig 2C, r = .31, p = .016; Spearman’s rho = .28, p = .034; consisting of the subscales “Exploratory Excitability”: r = .253, p = .051; rho = .26, p = .04; “Impulsiveness”: r = .289, p = .025; rho = .24, p = .066; “Extravagance”: r = .285, p = .027; rho = .22, p = .091; “Disorderliness”: r = .075, p = .57; rho = .07, p = .58). This correlation remains significant when controlling for the two other two PTQ scales (r = .269, p = .041), highlighting the specificity of novelty seeking. Exploratory analysis of the other TPQ scales revealed a significant correlation with the “Fear of uncertainty” subscale of the “Harm aversion” scale (r = -.36, p = .005; rho = -.36, p = .005).

Correlating the Novelty Seeking subscale with the RT facilitation for short and long delays separately revealed that the correlation between the RT effect and novelty seeking is driven by a correlation with the RT facilitation after long delay. Novelty seeking scores did not significantly correlate with the RT facilitation after short delays (“Novelty seeking”: r = .02, p = .85; Spearman’s rho = .09, p = .49; consisting of the subscales “Exploratory Excitability”: r = -.02, p = .85; rho = -.03, p = .82; “Impulsiveness”: r = -.03, p = .83; rho = .04, p = .771; “Extravagance”: r = -.04, p = .78; rho = .09, p = .51; “Disorderliness”: r = .19, p = .14; rho = .21, p = .11), while significant correlations were found with the RT facilitation after longer delays (“Novelty seeking”: r = .28, p = .028; Spearman’s rho = .26, p = .047; consisting of the subscales “Exploratory Excitability”: r = .32, p = .012; rho = .35, p = .006; “Impulsiveness”: r = .25, p = .058; rho = .18, p = .16; “Extravagance”: r = .26, p = .048; rho = .19, p = .15; “Disorderliness”: r = -.02, p = .9; rho = -.02, p = .89). While the correlation appears to be driven by the trials with long delays, note however that the strengths of the correlations of novelty seeking with the RT effect after short or long delay do not differ significantly from each other (Novelty Seeking: p = .22; Exploratory Excitability: p = .1). Additionally, note that the two bins do not include the same number of trails. Exploratory analysis of correlations of the RT facilitation after short or long delays with other TPQ scales revealed no significant correlations.

## Discussion

Long standing experimental evidence strongly suggests that novelty is intrinsically motivating and leads to exploration [34–37], a link that appears well conserved in animals and humans [38–40]. Our results now show that stimulus novelty enhances the speed of subsequent actions in humans in the absence of reward, as well as response accuracy. The time course of this enhancement shows that the effect is strongest when there is little time to prepare a response, akin to what might be considered impulsive choices. However, individual differences reveal that the trait novelty seeking is associated with a response facilitation after long delays, akin to vigour or sustained exploratory drive. We discuss these findings from the vantage point of exploration and approach, considering the functional anatomical organization of novelty processing within the hippocampus and basal ganglia. We also consider how the energizing effect of novelty could lead to suboptimal behaviour when novelty and the value of exploration are not aligned.

In our experiment, we measured the magnitude of the RT facilitation by novelty by comparing it to RTs elicited for familiar images. By doing so, we obtained a conservative estimate of the energizing effects of novelty. This is because familiarity with an image should make it easier to decode action requirements compared to an entirely novel image. Thus, in the absence of any motivational differences between novel and familiar images, the prediction would be that a novel image will slow down action selection and execution. From this perspective, the comparison to familiar images likely led us to underestimate the RT facilitation observed for novelty.

Manipulating the length of the delay between the novel or familiar cue and action requirements allowed us to investigate the time course of the energizing effects of novelty. Since the delay critically constrains the time available for decision making prior to action, it is a proxy for impulsivity at short delays and deliberate choice at long delays. By the same token, the fact that short delays showed a stronger RT improvement than long delays points towards a role of stimulus novelty in promoting impulsivity. The presence of an energizing effect for immediately required action is also consistent with the role of novelty on impulsive choice and approach actions [41–44]. The lack of an effect of novelty after long delays shows that the energizing effect of novelty decayed rapidly over time. However, we also found that novelty seeking correlated with RT enhancement by novelty after long delays. This suggests that novelty seeking prolonged the facilitating effect of novelty by several seconds. Understanding the circumstances in which novelty promotes actions could be of clinical relevance given the empirical link between novelty seeking and proneness to drug addiction [3,4,45–47]. To the extent that our task is a valid proxy for approach actions, our findings would indicate that novelty seeking would energize approach decisions even if there is opportunity for longer deliberation.

The temporally extended facilitation of action (Go responses after long delays) that we found in novelty seekers is conceptually compatible with reward-related vigour. In previous studies, vigour was operationalized as faster performance of instrumental responses related to the local average of the recent reward history [48] which might also be related to dopamine agonism [49]. Importantly, as in our study, this enhancement of reaction times had no instrumental impact per se.

We conjecture that the facilitation of actions in this task relies both on the hippocampal detection of novelty [9–13,50] and dopamine release by the SN/VTA [23,27,49]. Previous work in animals and humans highlights two possible pathways through which the hippocampus can control dopamine release by the SN/VTA. The hippocampus-SN/VTA loop projects novelty signals indirectly from the hippocampus by activating the nucleus accumbens which inhibits the ventral pallidum, in turn releasing inhibition on dopaminergic neurons in the SN/VTA [7,8]. Another relevant pathway in which dopaminergic neurons in the SN/VTA are activated by the hippocampus, specifically CA3, is relayed via the lateral septum [16]. This pathway is especially relevant given recent imaging results show that the human CA3 (together with the dentate gyrus) is activated by novel photographic images [13] similar to those used in our study. The noradrenergic locus coeruleus is another midbrain/brain stem structure that is activated by novelty and may be relevant for our findings because of its positive effects on arousal [51,52]. The locus coeruleus also regulates hippocampal synaptic plasticity in the context of spatial exploration [53].

A previous study on exploration showed that when making choices between rewarding options, stimulus novelty and associated activation of the hippocampus and the SN/VTA enhance the likelihood of choice for options imbued with stimulus novelty [6]. In that study, action was always required to express a choice between rewarding options. Therefore, it remained unclear the impact of stimulus novelty on pure approach actions without reward or competing rewards. In fact, animal studies indicate that novelty seeking can be related to approach towards unrewarding stimuli [42]. Rats that are high novelty seekers are more likely to show sign-tracking behaviour, in which a cue for a reward acquires incentive salience and is approached [42,43]. Sign-tracking is related to dopamine release and can be maladaptive because a cue that has no intrinsic value in itself is approached and indeed the approach to the actual reward is delayed due to this engagement with the cue [42]. In this case approach is targeted towards a stimulus that is not rewarding in itself. The present results show that stimulus novelty can energize action in the absence of explicit reward. It is possible that this form of energization shares mechanisms with sign-tracking.

The fact that we observed energization that is not instrumentally relevant is also akin to Pavlovian-instrumental transfer. In Pavlovian-instrumental transfer, the presence of a positively conditioned stimulus enhances the vigor for responses that are instrumentally independent of the displayed conditioned stimulus [54]. In the case of our experiment however, the stimulus is not conditioned with a reward expectation but merely by novelty itself. This adds to an argument for a likely conceptual and functional link between reward and novelty in motivation as reflecting a “hard-wired” effect [21,55].

Another parallel between novelty and reward is its positive impact on both reaction times and reaction accuracy at the same time. It has been shown that reward can improve performance without following a speed/accuracy trade-off [56–58]. The fact that novelty enhances both accuracy and reaction time in the current study is consistent with an effect similar to that of reward. This is consistent with a general positive effect on motivation, attention or control. An alternative account of the observed temporal effects could involve working memory and the maintenance of task instructions over an extended period, and therefore increasing difficulty or decay of attention.

The potential value of a novel stimulus cannot be scrutinized without exploration and approach. Indeed, the motivationally energizing effects of novelty have been accounted for in cognitive theories [40] and anatomical models [7] but have not been conclusively demonstrated in humans. Here, we uncover a rapid action bias induced by stimulus novelty that could potentially energize exploratory behaviours and approach. This bias (or “bonus”) appears to be “hard-wired” because it was not dependent on any outcome or prior learning. Individual differences in novelty seeking revealed that novelty seekers tend to have a more prolonged time window for energization/vigor following novelty. A link between action and stimulus novelty could be a simple mechanism supporting intrinsic motivation when rewards are sparse and, therefore, learning through reinforcement is difficult. Future studies could explore the energizing effect of novelty on more complex models of approach (and withdrawal), how it is dependent on the integrity of the hippocampus and the basal ganglia, and whether it declines in aging and incipient neurodegenerative conditions [59].

## Supporting Information

S1 Data Data Matrix. Dataset the analysis was based on.
(XLSX)
